# Validation of the Young Schema Questionnaire-Short Form in a Flemish Community Sample

**DOI:** 10.5334/pb.406

**Published:** 2018-04-23

**Authors:** Els Pauwels, Eva Dierckx, Dirk Smits, Rianne Janssen, Laurence Claes

**Affiliations:** 1 Faculty of Psychology and Educational Sciences, KU Leuven – University of Leuven, Tiensestraat 102, 3000 Leuven, BE; 2 Psychiatric Hospital Alexianen Tienen, Liefdestraat 10, 3300 Tienen, BE; 3 Odisee University College, Brussels, Warmoesberg 26, 1000 Brussels, BE; 4 Department of Clinical and Lifespan Psychology, Vrije Universiteit Brussel, Pleinlaan 2, 1050 Brussels, BE; 5 Faculty of Medicine and Health Sciences, University of Antwerp, Campus Drie Eiken, Wilrijk, BE

**Keywords:** Early Maladaptive Schemas, Validity, Reliability, Young Schema Questionnaire, Big Five

## Abstract

The Young Schema Questionnaire (YSQ, Young, 1994) was developed to assess Early Maladaptive Schemas (EMS), which account for the dysfunctional beliefs in individuals with personality disorders or maladaptive personality traits. This study aims to investigate the factor structure, the reliability and the validity of the original YSQ – Short Form (Young & Brown, 1998; YSQ-SF, 15 EMS) as well as an extension including 16 EMS, based on the 16 factor structure of the YSQL2 (Young & Brown, 1998) in a community sample. The sample consisted of 672 participants (51% females; *M*_age-total_ = 44.34; *SD*_age-total_ = 16.24). Results show evidence for both the 15 and 16 factor solution of the YSQ-SF with good internal consistency coefficients for the different scales. Significant gender differences were observed for Self-Sacrifice (females higher) and Entitlement (men higher), along with different patterns of correlations between age and Insufficient Self-Control (*r* = –.19), Enmeshment (*r* = –0.16) and Self-Sacrifice (*r* = .13). Furthermore, Big Five personality traits were significantly associated with several schema scales. In sum, we can conclude that both the YSQ-SF15 and 16 are valid instruments to assess EMS among a Flemish community sample. However, given the unique additive value of the 16^th^ EMS (Social Undesirability) in predicting lower scores on Extraversion and Openness, the use of the YSQ-SF16 could be favored.

## 1 Introduction

Schema theory is a theoretical framework developed by Jeffrey Young (Young, 1994). Central in this theory is the concept of Early Maladaptive Schemas (EMS). These EMS are considered to develop from negative childhood experiences into maladaptive core beliefs which are self-perpetuating and therefore (without appropriate treatment) rather resistant to change. As such EMS are maintaining factors for chronic psychopathology and more specific personality psychopathology.

In Young’s schema theory, the EMS are categorized into five higher-order schema domains representing unmet emotional needs in childhood: disconnection, impaired autonomy, impaired limits, other directedness and disinhibition (see Table 1). To assess these EMS, Young developed the Young Schema Questionnaire (YSQL2; Young, 1994), initially comprising 205 items and 16 EMS scales.

**Table 1:** Schema domains and corresponding schema scales (Young, 1990).

Schema domains	Schema scales

*Disconnection and Rejection*	Emotional Deprivation (ED), Mistrust/Abuse (MA), Abandonment (AB), Social Isolation (SI), and Defectiveness/Shame (DS)
*Impaired Autonomy and Performance*	Social Undesirability (SU), Failure to Achieve (FA), Dependence/Incompetence (DI), Enmeshment (EM), Vulnerability to Harm and Illness (VH)
*Impaired Limits*	Entitlement/Grandiosity (ET) and Insufficient Self-Control/Self-Discipline (IS)
*Other Directedness*	Subjugation (SB) and Self-Sacrifice (SS)
*Over-Vigilance and Inhibition*	Emotional Inhibition (EI) and Unrelenting Standards (US)

In 1998, Young and Brown constructed a shorter inventory by which 15 instead of the original 16 schemas were assessed, the YSQ-Short Form (YSQ-SF). By developing the YSQ-SF, Young (1998) shortened his original questionnaire (YSQL2) by reducing the number of items per schema to five items and by eliminating all items of the Social Undesirability schema. This short version was constructed partially based on an exploratory factor analysis performed on the long form (YSQL2) by Schmidt and colleagues (1995) and those results were in a later stage replicated by Lee and colleagues (1999). In these studies, only 15 out of the 16 scales emerged from the exploratory factor analyses. For each of the 15 schemas, the five highest loading items of each scale were selected in order to create the YSQ-SF.

Hoffart and colleagues (2005) confirmed the 15 factor structure by means of confirmatory factor analyses (CFA) in a mixed community and clinical sample of 1037 participants. Also other authors (e.g., Calvete et al., 2005; Lodoño et al., 2012) confirmed this 15 factor structure in large student samples (N = 407 and N = 1392 respectively) by means of CFA. However, studies in which the 16 factor structure of the YSQ was investigated are scarce and were only performed on the long version of the YSQ (Rijkeboer & Van den Bergh, 2005; Pauwels et al., 2013). To our knowledge, only Hoffart and colleagues (2005) investigated the 15 factor structure and the 16 factor model of the YSQ-SF (in which all of the 9 items of the Social Undesirability scale were included), both in a clinical and non-clinical sample (N = 1037). CFA results favoured the 15 factor structure of the YSQ-SF. However, adding 9 items to measure Social Undesirability (the 16^th^ EMS) may lead to an overrepresentation of the 16^th^ schema, as all other schemas were assessed by only 5 items. As far as we know, no one has created a 5-item shorter version of the Social Undesirability scale.

Additionally, several authors have investigated the reliability or internal consistency coefficients of all YSQ-SF scales and reported Cronbach’s alpha coefficients from good to very good (e.g., Baranoff et al., 2006; Cui et al., 2011; Oei & Baranoff, 2007; Welburn et al., 2002).

Concerning gender differences, Lodoño et al. (2012) found higher scores for men on all YSQ-SF scales except for Abandonment and Enmeshment (N = 1392; 39% males), whereas Lachenal-Chevallet et al. (2006) reported higher scores for men on Entitlement, Insufficient Self-Control, Emotional Deprivation and Mistrust compared to women in community samples (N = 263; 31% males). Concerning age differences, Nordahl, Holthe and Haugum (2005) reported that YSQL2 scale scores were positively associated with increasing age in outpatients. Similarly, Pauwels and colleagues (2013) showed that age was positively related to Emotional Deprivation and negatively related to Enmeshment in clinical inpatients. Additionally, when comparing younger, middle aged and older adults, Pauwels et al. (2014) found that younger adults tend to report higher scores on Insufficient Self-Control compared to middle and older adults; whereas older adults tend to have lower scores on Enmeshment compared to middle/younger adults.

The Diagnostic and Statistical Manual of Mental Disorders (5th ed.; DSM-5; American Psychiatric Association (APA), 2013) offers an alternative, dimensional model of Personality Disorders in which personality disorders are described by several maladaptive personality traits. Dimensional personality trait models state that combinations of adaptive and maladaptive personality traits underlie personality disorders (Widiger et al. 2009). Focusing on convergent validity of the YSQ, many studies investigated the association between YSQ scales and maladaptive personality traits/disorders in both non-clinical and clinical samples (e.g., Bach et al., 2016; Pauwels et al., 2013), while only a limited number of studies have investigated the link between the EMS scores and adaptive personality traits like the Big Five personality traits, one of the most known dimensional personality trait models (McCrae & Costa, 2003). When comparing the few studies investigating the relationship between the Big Five personality traits and the EMS scores within community samples, results showed positive associations between Neuroticism and most EMS. Extraversion was positively associated with Unrelenting Standards and negatively with all other EMS. Openness was positively related to Vulnerability to Harm and Unrelenting Standards; whereas Agreeableness were positively associated with Self-Sacrifice and Unrelenting Standards and negatively with Emotional Deprivation, Mistrust, Social Isolation and Emotional Inhibition. Finally, Conscientiousness was positively associated with Unrelenting Standards (Bahrami Ehsan & Bahramizadey, 2011; Bahramizadeh & Bahrami Ehsan, 2011; Muris, 2006, Sava, 2009; see Table 2 for a detailed overview).

**Table 2:** Literature associations between EMS and Big Five personality traits.

Study	Sample	Method	Results

**Muris (2006)**	N = 173 students 50% females Mean age = 13	CORRELATIONS (corrected for gender/age) YSQ-A (adolescent version) Big Five Questionnaire for Children	Neuroticism = ↑ all EMS Extraversion = ↑US Openness = ↑ VH and ↑ US Agreeableness = ↑ SS and ↑ US Conscientiousness = ↑ US
**Sava (2009)**	N = 154 students 56.5% females Median age = 21	CANONICAL CORRELATIONS YSQ-L2 (long version) DECAS Personality Inventory	I: ↓ Agreeableness and ↑ Neuroticism = ↑ all Schema domains II: ↑Neuroticism = ↑ Impaired Autonomy and ↑ Other Directedness
**Bahrami Ehsan and Bahramizadeh (2011)**	N = 200 students 50% female Mean age = 24.62	CORRELATIONS YSQ-SF (short version) NEO-FFI (Big Five inventory)	Agreeableness = ↓ ED, MA, SI and EI
**Bahramizadeh and Bahrami Ehsan (2011)**	N = 200 students 50% female Mean age = 24.62	CORRELATIONS YSQ-SF NEO-FFI (Big Five inventory)	Neuroticism = ↑ all EMS Extraversion = ↓ all EMS, except for ET and SS
**Thimm (2010)**	N = 147 outpatients 74% females Mean age = 39.2	BIVARIATE CORRELATIONS **SEMIPARTIAL CORRELATIONS**	Neuroticism = ↑ most EMS (not SS and ET) **Neuroticism =** ↑ **most EMS (not SS, ET, ED, EM, EI)** Extraversion = ↓ ED, MA, **SI**, FA, DS, SB, **EI** Openness = ↓ FA and EI Agreeableness = ↑ SS and ↓ MA, **ET** and **IS** Conscientiousness = ↓ DI and IS **Conscientiousness =** ↑ **US and** ↓ **IS**

*Note.* ↑ = positive correlation; ↓ = negative correlation; EMS = Early Maladaptive Schema; Emotional Deprivation (ED), Mistrust/Abuse (MA), Abandonment (AB), Social Isolation (SI), and Defectiveness/Shame (DS); Social Undesirability (SU), Failure to Achieve (FA), Dependence/Incompetence (DI), Enmeshment (EM), Vulnerability to Harm and Illness (VH); Entitlement/Grandiosity (ET) and Insufficient Self-Control/Self-Discipline (IS); Subjugation (SB) and Self-Sacrifice (SS); Emotional Inhibition (EI) and Unrelenting Standards (US); YSQ = Young Schema Questionnaire.

In sum, existing research investigating EMS differences in personality traits, was mostly based on the YSQ-SF with 15 factors. From a clinical point of view, it would be interesting to investigate the additional value of the 16^th^ EMS in a shorter version of the questionnaire given the proven additional value of this 16^th^ factor in the longer version of the YSQ (Pauwels et al., 2013; Rijkeboer & van den Bergh, 2006) in understanding for example eating disorders and cluster C psychopathology. Therefore, the first aim of the present study was to replicate the existing 15 factor structure of the YSQ-SF in a community sample. The second aim was to shorten the 16^th^ factor ‘Social Undesirability’ into 5 items, in line with the other EMS. The third aim was to investigate the psychometric properties of the expanded YSQ-SF16 (the original YSQ-SF15 + additional schema), by investigating the reliability of the YSQ-SF16 scales, age and gender differences as well as the associations of the YSQ-SF16 with the Big Five personality traits.

## 2 Method

### 2.1 Participants and procedure

Six hundred seventy two participants were included in this study. The sample consisted of 328 males (48.4%; *M*_age_ = 43.81; *SD*_age_ = 16.44) and 344 females (51.2%; *M*_age_ = 44.85; *SD*_age_ = 16.05) with no significant age differences. Almost 18% (17.6%) of the sample had a master’s degree, 16.7% a bachelor degree and 65.8% did not have higher education qualification. Participants were sampled by students, who were asked to select participants according to a predetermined set of variables (e.g., a male between 25–30 years old with a higher level of education), in order to gain course credits. This set of variables was based on the age, gender and education distribution of the Flemish population between 18 and 75 years of age. All participants of the study gave a written informed consent before participating in the study and all participants were given an access code to fill out a secured web-based survey. The ethical committee of the KU Leuven (SMEC) approved the study.

### 2.2 Instruments

The Young Schema Questionnaire-Long Form was used to assess the EMS (YSQL2; Young & Brown, 1994; Dutch version: Young & Pijnaker, 1999) and consists of 205 items, which are divided over 16 subscales corresponding with the 16 EMS scales. From this long – 205 items – version, the 75 items of the YSQ-SF15 (Young & Brown, 1998) and the 9 items of the Social Undesirability schema were selected. Table 1 shows a summary of the 16 EMS and their corresponding schema domains. The items are rated on a 6-point Likert scale ranging from 1 “*Completely untrue for me*” to 6 “*Describes me perfectly*.” The Dutch version shows good psychometric properties comparable to existing research (e.g., Lee et al., 1999; Schmidt et al., 1995; Pauwels et al., 2013).

To assess the Big Five personality traits, the Big Five Inventory (BFI-25, John & Srivastava, 1999, shortened by Boele, Sijtsema, Klimstra, Denissen & Meeus, 2017) was used. The BFI-25 consists of 25 items and measures the Big Five personality dimensions: Neuroticism, Extraversion, Openness to Experience, Agreeableness and Conscientiousness. Items are answered on a 5-point Likert scale ranging from 1 indicating ‘strongly disagree’ to 5 ‘strongly agree’. The Dutch version shows good psychometric properties comparable to the English version of John and Srivastava (1999). In our sample Cronbach alpha values range from .65 (Agreeableness) to .81 (Extraversion) indicating moderate to good levels.

### 2.3 Data analyses

*Adaptation of the YSQ-short form 16 (YSQ-SF16).* The short version of the YSQ was obtained by selecting the 75 items of the internationally validated YSQ-SF15 (Young & Brown, 1998). This short form consists of 75 items divided across 15 EMS, i.e., all EMS of the YSQL2 except for Social Undesirability (SU). In order to create a similar scale for the SU EMS, the 5 highest loading items based on a factor analysis on all 9 SU items of the YSQL2 were selected.

*Factor structure of the YSQSF15 and YSQSF16.* Confirmatory factor analyses (CFA) was used to investigate the latent structure of the YSQ-SF15 and YSQ-SF16. CFA-model parameters were estimated by a Weighted Least Squares Means and Variances Adjusted estimation method (WLSMV option, MPLUS 7.4, Muthen & Muthen, 2015), as the response scale is categorical. Multiple fit statistics were used to gauge the model fit (Hu & Bentler, 1998, 1999), with a Root-Mean squared error of approximation (RMSEA) close to or below .06 and a comparative fit index (CFI) close to or higher than .95 indicating a good fit (RMSEA < .09, CFI > .90 for acceptable fit). In a first step, the 15-factor model was fitted (YSQ-SF15). Second, it was investigated whether the addition of a 16^th^ EMS (i.e., SU) influenced the fit of our model (YSQ-SF16).

*Internal Consistency of the YSQ-SF15/16.* Cronbach alpha coefficients were calculated for the 15/16 scales of the YSQ-SF15/16. Alpha coefficients above .70 are considered to be adequate, alpha values of .80 and more are considered to be good to very good (Nunnally & Bernstein, 1994).

Gender and age differences for the YSQ-SF15/16 scales were analyzed. To investigate gender differences a MANOVA with gender as a fixed factor and the sum of the YSQ scales as dependent variables were used. To assess effect size, partial eta squared was used, with a value of .01 indicating a small effect, .06 a medium effect and .13 a large effect. The associations between age and the YSQ-SF15/16 scales were studied by means of the Pearson correlation coefficients. A Bonferroni correction was applied to control the family wise error rate (.05/16 = .003). Effect sizes were based on the percentage of common variance explained: >10% common variance for practical importance (correlation > .224); 20%–30% for medium effect (correlation between .447–.548) and strong effect > 50% (correlation > .707) (Cohen, 1992).

Associations between the YSQ-SF16 scales and BFI-25 scales were calculated by means of multiple stepwise linear regression analyses using SPSS 24. The 5 BFI-25 sum scores were entered as dependent variables in five different regression analyses. Gender and age were entered in the first block as control variables and the 16 YSQ-SF16 sum scores were entered stepwise in the second block as predictors. R² change of the YSQ-SF16 schema scales was examined to look for the unique predictors of the 5 BFI-25 scales). A Bonferroni correction was applied to control the family wise error rate (*p* < .05/5 = .01).

## 3 Results

*Adaptation of the EMS scale Social Undesirability (SU) of the YSQ-SF16.* Factor analysis indicated that item 70 (I’m not sexually attractive.), item 73 (I can’t carry on a decent conversation.), item 74 (I’m dull and boring in social situations.), item 76 (I never know what to say socially.) and item 77 (People don’t want to include me in their groups.) showed the highest factor loadings on the SU scale. The YSQ-SF16 therefore consists of 80 items: i.e., the 75 items of the YSQ-SF15 in combination with the items 70, 73, 74, 76 and 77 (SU scale).

*Construct validity of the YSQ-SF15/16.* Based on the fit criteria, a 15 factor model (YSQ-SF15) fits our data well: *Chi²* = 5147.163; *df* = 2595; RMSEA = .038; CFI = .954. The model with the additional SU factor (YSQ-SF16) also obtained a good fit to the data: *Chi²* = 5733.482; *df* = 2960; RMSEA = .037; CFI = .954. In Table 3, loadings of the items on their schemas are presented for the final 16 factor model. Except for item 167, all loadings exceed .40. Factor correlations are shown in Table 4 and varied from .11 to .85.

**Table 3:** Factor Loadings 16 factor model for YSQ-SF.

	ED	AB	MA	SI	DS	SU	FA	DI	VH	EM	SB	SS	EI	US	ET	IS

**YSQ4**	.724															
**YSQ6**	.852															
**YSQ7**	.851															
**YSQ8**	.946															
**YSQ9**	.829															
**YSQ11**		.768														
**YSQ12**		.840														
**YSQ17**		.712														
**YSQ18**		.868														
**YSQ25**		.873														
**YSQ28**			.695													
**YSQ30**			.915													
**YSQ32**			.886													
**YSQ35**			.820													
**YSQ37**			.790													
**YSQ45**				.882												
**YSQ46**				.694												
**YSQ47**				.898												
**YSQ48**				.915												
**YSQ50**				.911												
**YSQ55**					.913											
**YSQ56**					.885											
**YSQ59**					.941											
**YSQ60**					.908											
**YSQ61**					.942											
**YSQ70**						.846										
**YSQ73**						.843										
**YSQ74**						.906										
**YSQ76**						.768										
**YSQ77**						.931										
**YSQ79**							.919									
**YSQ80**							.977									
**YSQ81**							.874									
**YSQ83**							.897									
**YSQ84**							.873									
**YSQ88**								.891								
**YSQ93**								.714								
**YSQ97**								.841								
**YSQ98**								.898								
**YSQ99**								.919								
**YSQ103**									.906							
**YSQ104**									.738							
**YSQ106**									.660							
**YSQ109**									.829							
**YSQ110**									.726							
**YSQ117**										.797						
**YSQ118**										.726						
**YSQ119**										.852						
**YSQ121**										.925						
**YSQ122**										.900						
**YSQ129**											.867					
**YSQ130**											.891					
**YSQ131**											.746					
**YSQ132**											.888					
**YSQ135**											.866					
**YSQ141**												.849				
**YSQ143**												.590				
**YSQ148**												.804				
**YSQ149**												.510				
**YSQ151**												.748				
**YSQ159**													.716			
**YSQ160**													.771			
**YSQ161**													.829			
**YSQ162**													.774			
**YSQ163**													.762			
**YSQ164**														.699		
**YSQ167**														.422		
**YSQ170**														.292		
**YSQ171**														.820		
**YSQ176**														.831		
**YSQ180**															.696	
**YSQ182**															.860	
**YSQ183**															.708	
**YSQ184**															.814	
**YSQ185**															.673	
**YSQ192**																.681
**YSQ194**																.780
**YSQ195**																.774
**YSQ201**																.753
**YSQ203**																.848

*Note.* Emotional Deprivation (ED), Mistrust/Abuse (MA), Abandonment (AB), Social Isolation (SI), and Defectiveness/Shame (DS); Social Undesirability (SU), Failure to Achieve (FA), Dependence/Incompetence (DI), Enmeshment (EM), Vulnerability to Harm and Illness (VH); Entitlement/Grandiosity (ET) and Insufficient Self-Control/Self-Discipline (IS); Subjugation (SB) and Self-Sacrifice (SS); Emotional Inhibition (EI) and Unrelenting Standards (US).

**Table 4:** Interfactor correlations between EMS.

	ED	AB	MA	SI	DS	SU	FA	DI	VH	EM	SB	SS	EI	US	ET	IS

**ED**	1															
**AB**	.617	1														
**MA**	.662	.723	1													
**SI**	.667	.598	.703	1												
**DS**	.675	.717	.706	.829	1											
**SU**	.586	.648	.623	.822	.872	1										
**FA**	.527	.611	.554	.621	.721	.768	1									
**DI**	.589	.716	.664	.720.	849	.828	.807	1								
**VH**	.447	.670	676	.596	.659	.609	.601	.730	1							
**EM**	.129	.605	.600	.603	.664	.650	.598	.760	.677	1						
**SB**	.526	.702	.661	.660	.732	.768	.729	.836	.668	.778	1					
**SS**	.254	.308	.359	.159	.176	.203	.206	.211	.250	.252	.429	1				
**EI**	.587	.615	.670	.732	.733	.767	.559	.698	.602	.588	.698	.368	1			
**US**	.246	.403	.457	.445	.410	.384	.257	.376	.400	.399	.442	.402	.590	1		
**ET**	.293	.384	.499	.459	.446	.340	.217	.410	.390	.444	.410	.179	.517	.641	1	
**IS**	.391	.560	.494	.604	.637	.630	.625	.691	.550	.560	.638	.114	.641	.384	.589	1

*Note.* Emotional Deprivation (ED), Mistrust/Abuse (MA), Abandonment (AB), Social Isolation (SI), and Defectiveness/Shame (DS); Social Undesirability (SU), Failure to Achieve (FA), Dependence/Incompetence (DI), Enmeshment (EM), Vulnerability to Harm and Illness (VH); Entitlement/Grandiosity (ET) and Insufficient Self-Control/Self-Discipline (IS); Subjugation (SB) and Self-Sacrifice (SS); Emotional Inhibition (EI) and Unrelenting Standards (US).

*Internal Consistency of the YSQ-SF15/16.* Cronbach alpha values were calculated for the 16 scales of the YSQ-SF15/16. Alpha values ranged from .76 (Unrelenting Standards) to .92 (Defectiveness/Shame and Failure to Achieve) indicating adequate to good internal consistency of the 15 YSQ-SF15 scales. The newly created SU scale showed an internal consistency of .88 (Table 5).

**Table 5:** Means (standard deviations) of EMS scores across gender.

	Total Sample *M (SD)*	α	Females *M (SD)*	Males *M (SD)*	*F* (16,655)	Effect size partial η^2^	Range

***Disconnection and Rejection***							
Emotional Deprivation	9.90 (5.38)	.88	9.76 (5.66)	10.04 (5.07)		0.001	[5, 30]
Abandonment	9.91 (4.91)	.85	10.24 (5.21)	9.57 (4.56)		0.005	[5, 30]
Mistrust/Abuse	10.03 (4.80)	.87	9.79 (4.88)	10.29 (4.72)		0.003	[5, 30]
Social Isolation	8.86 (4.76)	.89	8.44 (4.67)	9.30 (4.82)		0.008	[5, 30]
Defectiveness/Shame	7.06 (3.6)	.92	6.94 (3.82)	7.18 (3.38)		0.001	[5, 30]

***Impaired Autonomy and Performance***							
Social Undesirability	8.55 (4.39)	.88	8.48 (4.56)	8.62 (4.21)		0.000	[5, 30]
Failure to Achieve	9.28 (4.96)	.92	9.45 (5.09)	9.10 (4.83)		0.001	[5, 30]
Dependence/Incompetence	7.61 (3.59)	.85	7.53 (3.83)	7.70 (3.31)		0.001	[5, 30]
Vulnerability to Harm	8.76 (4.10)	.81	8.92 (4.24)	8.59 (3.94)		0.002	[5, 26]
Enmeshment	7.25 (3.44)	.84	7.32 (3.56)	7.17 (3.30)		0.000	[5, 30]

***Impaired Limits***							
Entitlement/Grandiosity*	10.66 (4.40)	.81	9.83 (3.92)	11.54 (4.70)	26.06***	0.037	[5, 28]
Insufficient Self-Control/Self-Discipline	10.76 (4.63)	.83	10.31 (4.34)	11.24 (4.88)		0.010	[5, 28]

***Other Directedness***							
Subjugation	9.05 (4.57)	.89	9.26 (4.91)	8.82 (4.19)		0.002	[5, 30]
Self-Sacrifice*	15.26 (5.14)	.81	16.28 (5.25)	14.20 (4.81)	28.45***	0.041	[5, 30]

***Over-Vigilance and Inhibition***							
Emotional Inhibition	10.41 (4.79)	.82	9.96 (4.65)	10.88 (4.89)		0.009	[5, 30]
Unrelenting Standards	14.62 (4.97)	.76	14.46 (5.16)	14.79 (4.77)		0.001	[5, 30]

*significant at p < .003. *Note*: *p ≤ .05, **p ≤ .01, ***p ≤ .001; Effect size: partial η² = .01 for a small effect, partial η² = .06 for a medium effect, and partial η² = .13 for a large effect.

For gender differences, the results of the MANOVA with gender as independent variable and the YSQ-SF16 scales as dependent variables, showed significant differences for two scales (Wilks λ = .87, *F*(16, 655) = 5.96, *p* < .003; partial η² = .13) (Table 5). Women scored significantly higher on Self-Sacrifice, whereas men scored significantly higher on Entitlement, although effect sizes were small (<.06). For age, significant correlations between age and the YSQ scales were found for three schema scales. Age was significantly negatively correlated with Enmeshment (*r* = –0.16, *p* < .003) and Insufficient Self-Control (*r* = –.19, *p* < .003) and positively with Self-Sacrifice (*r* = .13, *p* < .003) although none of the effect sizes reached the level of practical relevance (<.22). Given the significant differences in EMS scores for gender and age, it will be important to control for gender and age differences when investigating the associations between the EMS scales and Big Five personality traits.

Five multiple regression analyses were conducted to see which EMS scales were significantly related to the Big Five personality dimensions after controlling for age and gender. Results in Table 6 show that Neuroticism was significantly positive related to high scores on Abandonment, Emotional Inhibition, Vulnerability to Harm and Insufficient Self-Control and negatively related to Entitlement. Extraversion was significantly negatively related with Social Undesirability and Emotional Inhibition, but positively related with Defectiveness/Shame and Unrelenting Standards. Openness was significantly negative related to Social Undesirability and positive with Unrelenting Standards and Social Isolation. Agreeableness was positively related with Self-Sacrifice, Unrelenting Standards and Subjugation and negatively related with Defectiveness/Shame, Emotional Inhibition, Entitlement and Mistrust. Finally, Conscientiousness was significantly negative related with Insufficient Self-Control, Failure to Achieve and Subjugation; whereas it was positively related with Unrelenting Standards and Emotional Inhibition.

**Table 6:** Multiple Regression for Big Five personality traits.

	Predictor	β	Λ R^2^

**Neuroticism**	Block1: Age Gender Block 2: Abandonment Emotional Inhibition Vulnerability to Harm Entitlement Insufficient Self-Control **R² = .28**	–.03 .26*** .22 .16 .12 –.14 .11	.08 .20

**Extraversion**	Block1: Age Gender Block 2: Social Undesirability Emotional Inhibition Defectiveness/Shame Unrelenting Standards **R² = .30**	–.04 .01 –.51 –.26 .21 .13	.00 .30

**Openness**	Block1: Age Gender Block 2: Social Undesirability Unrelenting Standards Social Isolation **R² = .14**	–.05 –.09* –.45 .17 .19	.02 .13

**Agreeableness**	Block1: Age Gender Block 2: Defectiveness/Shame Self-Sacrifice Emotional Inhibition Entitlement Mistrust/Abuse Unrelenting Standards Subjugation **R² = .31**	.07* .02 –.19 .27 –.26 –.20 –.15 .11 .12	.04 .27

**Conscientiousness**	Block1: Age Gender Block 2: Insufficient Self-Control Unrelenting Standards Failure to Achieve Emotional Inhibition Subjugation **R² = .43**	.16*** .08** –.50 .30 –.16 .14 –.09	.07 .36

*p < .05; **p < .01; ***p < .001 for age and gender; *p* < .01 for schema scales.

## 4 Discussion

In the present study, we were able to replicate the 15 as well as the 16 (including the SU schema) factor structure of the YSQ-SF in a Flemish community sample. The replication of the factor structure of the YSQ-SF15 reached good congruence coefficients (between .93 and .99) as compared with the YSQ-SF15 of Welburn and colleagues (2002). All EMS scales reached adequate reliability or internal consistency coefficients ranging from good to very good, comparable to previous studies (Oei & Baranoff, 2007).

Concerning gender differences, our results indicated that women scored significantly higher on Self-Sacrifice, whereas men reached significantly higher scores on Entitlement with small effect sizes (<.06). However, these results seem to indicate that women tend to be more focused on the needs of significant others; whereas men tend to be more focused on achieving their own goals and tend to feel more entitled to special rights as compared to women. The gender differences in our study were less pronounced as compared to the gender findings in other community samples (e.g., Lachenal-Chevallet et al. 2006; Lodoño et al., 2012). This difference can be due to the fact that the latter studies were performed in students samples with a smaller age range (*M*_age_ = 22.6; *SD*_age_ = 5 and *M*_age_ = 28; *SD*_age_ = 14) as compared to the current study. Gender differences in EMS in clinical samples on the other hand, seem to be less pronounced. This can be due to the fact that gender differences in clinical samples can also be influenced by gender differences in the prevalence of psychopathology (Pauwels, et al., 2013). With respect to age, we found that Enmeshment and Insufficient Self-Control decrease with increasing age, with small effect sizes (<.22). Similar findings for Enmeshment and Insufficient Self-Control were also found by Pauwels and colleagues (2014) in a clinical sample. Low scores on Enmeshment imply a certain amount of emotional stability and several studies indicate an improvement of emotional stability with increasing age (e.g., Carstensen et al., 2003). High scores on Insufficient Self-Control are often accompanied with more impulsivity and several studies indicate a decrease in impulsivity with increasing age (e.g., Segal, et al., 2006). Furthermore, the current study shows that Self-Sacrifice increases with age, which can be due to the changing importance of social relationships above the need to accomplish personal goals at older age. This change from intra- to interpersonal values is described within the socio-emotional selectivity theory of Carstensen et al. (1999).

With respect to convergent validity, the associations between the maladaptive EMS and the adaptive Big Five personality traits revealed more fine grained results compared to previous studies (e.g. Bahramizadeh & Bahrami Ehsan, 2011; Muris, 2006). Neuroticism, for example, was positively associated with several EMS, being: Abandonment, Emotional Inhibition, Vulnerability to harm and Insufficient Self-Control. These EMSs are also related to the Borderline Personality Disorder and the Avoidant Personality disorder (Pauwels et al., 2013; Samuel & Widiger, 2008). Extraversion was only negatively related with Social Undesirability and Emotional Inhibition, which are both positively related to Introversion (and thus negatively to Extraversion); the positive associations with Defectiveness/Shame and Unrelenting Standards are less clear. Additionally, Openness was negatively related to Social Undesirability, indicating low levels of self-esteem and positively with Unrelenting Standards or being eager to learn (McCrae & Costa, 2003), which was in line with the findings of Muris (2006). Agreeableness was positively related to Self-Sacrifice and Subjugation and negatively with Defectiveness/Shame, Mistrust and Entitlement which was in line with findings of other authors (Bahrami Ehsan, & Bahramizadeh, 2011; Muris (2006); Thimm, 2010). Agreeable people prefer social engagement above personal accomplishment and have a positive attitude toward the significant others. This pattern of results is also similar to people with dependent personality features (McCrae & Costa, 2003). Finally, Conscientiousness was negatively related to Insufficient Self-Control, thus indicating that conscientious people are self-disciplined and in line with the high scores on Unrelenting Standards (perfectionism) and low scores on Failure to Achieve (Stoeber, Otto, & Dalbert, 2009), and with features of the obsessive-compulsive PD, confirming the findings of Muris (2006) and Thimm (2010). Concerning the Social Undesirability schema, this schema seems to be of particular relevance for clinical samples (e.g. for Cluster C psychopathology in Pauwels et al., 2013), but also adds to the understanding of adaptive personality (e.g. high scores on Social Undesirability are associated with low scores on Openness and Extraversion). The different associations between EMS and both adaptive and maladaptive traits, may add to the dimensional trait models underlying personality disorders (Widiger et al., 2009).

Besides the strengths of the study, some limitations need further discussion. First, the factor structure of the YSQ-SF16 was established in a community sample. However in order to achieve good validity of the questionnaire, it can be important to consider measurement invariance of the questionnaire for age and gender. Future research should replicate this 16 factor structure in clinical sample as well. Second, the study was solely based on self-report questionnaires whereas sometimes EMS may be unconscious. Future studies should also include indirect measures of EMSs (Young & Brown, 2003). Third, caution to generalizing the results of the relationship between EMS and the Big Five personality traits is warranted for two reasons. First, results could be biased because BFI-25 scores were not corrected for acquiescence (Rammstedt & Farmer, 2013) and second, only few studies have investigated the relationship between adaptive Big Five personality traits and EMS. Nevertheless different alternative personality models of the DSM-5 state that combinations of both adaptive and maladaptive personality traits underlie personality disorders (Widiger et al. 2009).

In sum our study indicates that we were able to validate the factor structures and establish the psychometric features of the YSQ-SF15 as the YSQ-SF16 among a Flemish community sample. Gender and age differences, in our study, were less pronounced compared to findings of other studies and should be replicated in the future. Additionally, age and gender differences in community samples, seem to be less pronounced compared to gender and age differences in clinical samples, probably due to specific features of psychopathology. Furthermore, the Social Undesirability schema has a unique additional value in predicting low scores on Extraversion and Openness, which could favor the choice for the use of the YSQ-SF16 above the YSQ-SF15. Future studies in clinical samples, need to be performed to see whether these shorter YSQ-SF15/16 versions can be validated and applied in clinical samples, given their more user-friendly format.
